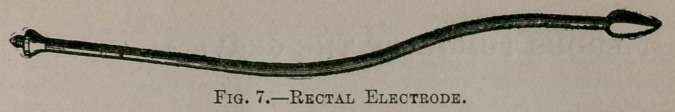# Electrolysis in Dermatology and in Some Other Departments of Practical Surgery

**Published:** 1888-07

**Authors:** George H. Rohé

**Affiliations:** Professor of Dermatology and Hygiene in the College of Physicians and Surgeons, Baltimore, Maryland


### ELECTROLYSIS IN DERMATOLOGY AND IN SOME OTHER DEPARTMENTS OF PRACTICAL SURGERY.

BY GEORGE H. ROHE, M. D.,

Professor of Dermatology and Hygiene in the College of Physicians and Surgeons, Baltimore, Maryland.

REMARKS MADE AT THE ANNUAL MEETING OF THE MEDICAL ASSOCIATION OF GEORGIA, APRIL 19, 1888.

In discussing a new method of treatment we should endeavor to divest ourselves of undue prepossession in its favor as well as of unreasoning prejudice against it. The antiseptic system in surgery and its logical foundation—the germ theory—which are now almost unreservedly accepted by the entire profession, were but a few years ago received with derision, and the battle-cry of the reactionaries, "Fort mit dem Spray," was thought to have driven the champions of the new surgery from the field. But results, by which the public always judges, resist all argument, and few surgeons at the present day dare to ignore the results obtained by the rigid application of the antiseptic system.

All advances in science and art are met at first with indifference, ridicule or depreciation. A rational conservatism is commendable, and the weapons just mentioned are perhaps justifiably used to prevent hasty action; but conservatism should not be allowed to become so ingrained as to be a bar to all progress; otherwise it degenerates into retrogression, which is as injurious in science as Bourbonism is in politics.

The method of treatment which I venture to bring to your attention, namely, Electrolysis, is at this time passing through the preliminary storm and stress period which seems to be the necessary precedent to acceptance by the profession. In some of its applications it has won for itself a place which, I venture to predict, it will maintain unshaken. In others it is still on trial, and the last word cannot yet be spoken for or against its usefulness. I shall endeavor to place before you fairly its just claims, and ask for them your careful consideration.

Electrolysis may be defined as the decomposition of a liquid into its constituent elements, the oxygen or electro-negative element being given off or collecting at the positive electrode, while the hydrogen or electro-positive element collects at the negative electrode.* The discovery of the electrolytic decomposition of water was made by Carlisle and Nicholson in the first year of the present century, but the conditions under which the process takes place, and the laws governing it, were most thoroughly studied by Faraday. To the latter observer we owe most of the definite knowledge available upon the subject.

The demonstration of the electrolysis (electrical analysis) of water was soon followed by the discovery that solutions of various salts, such as sulphate of copper, iodide of potassium, etc., could be decomposed in a similar manner by the electric current, and later it was found that organic tissues could likewise be resolved into their constituent elements. The exact manner in which this "organic analysis" is brought about is still under discussion, but the *fact* remains that it can be accomplished.

Without going into the theoretical aspects of the question, I will briefly recount some of the practical applications of this remedy, or method of treatment, limiting my remarks principally to the results obtained by my own experience.

The battery required is one that will furnish a constant current of sufficient quantity and intensity to overcome the interposed resistance. The zinc-carbon batteries, made by Waite & Bartlett, Flemming, or McIntosh, will answer the purpose very well, if kept

*Oxygen, chlorine, iodine, bromine and nitrogen are electro-negative elements, while hydrogen and the metals are electro-positive.

in good order and charged with properly prepared exciting fluid. I have seen a good battery ruined by pouring sulphuric acid into the cells, under the impression that the fluid had grown weak and needed to be fortified. It may as well be understood first as last that the physician who is unwilling to study the management of his battery, or who will not take some (often a good deal of) trouble to keep it in order, will have poor satisfaction in the use of electricity for any purpose.

In default of one of the portable batteries mentioned, from ten to twenty cells of a zinc-copper battery, or of the Léclanché pattern, will serve. The battery which I have used for several years for all sorts of electrolytic operations is a modification of the Daniell cell, known as the "Siemens-Halske." It is not portable, and is hence open to a serious objection, but in other respects it has no superior. It furnishes a perfectly constant current, is not liable to get out of order and requires very little attention. Of course it is understood that a constant current, or so-called galvanic battery, is meant. A Faradic battery will not answer for electrolysis.

In medical and surgical practice electrolysis is used with the following objects:

### I. REMOVAL OF SUPERFLUOUS HAIRS.

Drs. Michel and Hardaway, of St. Louis, first used electrolysis for the permanent removal of hairs growing in abnormal situations. The first named employed it successfully in relieving that troublesome malady, ingrowing eyelashes, while the latter extended its usefulness in the domain of dermatology. I have used the method since 1882 for the destruction of superfluous hairs, and believe it to be the only practical means at our command for this purpose. Of its entire success when properly employed there can be no longer any doubt. In addition to a proper battery, there will be needed for the treatment of a case of hypertrichosis the following qualifications, instruments and appliances:

1. A plentiful stock of patience.
2. A steady hand.
3. Good eyesight.

4. Proper electrodes.
5. A pair of cilia forceps.
6. A chair with head-rest.

Patience, a steady hand and good vision are essential qualifications for success.

For the positive electrode, the ordinary sponge-covered disk will answer. A needle-holder and fine needle will be required for the negative electrode.

The holder shown in the cut was devised by Dr. Hardaway and is made by the A. M. Leslie Company, of St. Louis. It is very convenient. A fine steel sewing needle (No. 12) may be used, but flexible needles made of an alloy of platinum and iridium are preferable. They are thinner than the finest steel needles, never break and can be bent into any shape desired.*

FIG. 1.—HARDAWAY'S NEEDLE-HOLDER.

Needle-holders are sometimes made with a key to make and break the circuit, but I do not think this an advantage. The needle-holder is attached to the conducting wire from the negative pole of the battery.

The forceps should have an easy spring, with flat, lightly serrated jaws and should not have a catch.

A chair with a firm head-rest must be used. I use an ordinary cane-seat arm-chair with adjustable head-rest, and find that it answers the purpose as well as a more complicated or expensive oculist's or dentist's chair.

The steps of the operation are as follows:

The patient is placed before a good light—avoiding direct sunlight unless modified by frosted glass—and directed to take hold of the handle of the sponge electrode, the sponge, of course, having been previously moistened. The operator then sits a little in front of and to the right of the patient and takes the needle electrode in his right hand, holding a pair of tweezers with flat, narrow jaws in his left. The needle is then gently insinuated into a follicle by the

*The needles are also manufactured by the A. M. Leslie Company, of St. Louis.

side of the hair until the bottom of the follicle is reached. This is manifested by a slight resistance to the onward passage of the needle. The patient is then directed to touch the sponge with the other hand, thus closing the circuit. The current will immediately pass and the electrolytic action be made manifest by a little frothing around the needle. In some skins also a little wheal will be raised about the follicle. In from twenty to forty seconds the hair can be extracted with the tweezers without the slightest resistance or pain. If the hair does not come away with perfect ease, the papilla has not been destroyed and the needle should be permitted to remain and the current to pass a little longer. The current is broken by removing the hand from the sponge electrode. This gives less pain than if the current is closed and opened with the needle. A current from one-half to two milliampères is sufficient.

If the hairs are very close together, they should not all be removed at the same time. The hairs should be picked out here and there; otherwise the points of irritation will be in too close proximity, and, if sufficiently intense, may produce small areas of sloughing and leave scars. If the operation is properly performed no visible scars should remain.

A sitting may last from fifteen to thirty minutes. Very few operators can extend it beyond the latter time. The sittings may be repeated every other day, or, in cases where time is important, every day.

After the operation a mild astringent lotion may be applied, and the patient should be directed to bathe the surface operated upon several times a day with hot water for five or ten minutes at a time. This tends to reduce any hyperæmia which may have been caused by the operation.

When the hair papilla has been thoroughly destroyed the hair cannot be regenerated. In most cases, however, a number of the hairs return, showing that the destruction of the papillæ has not been complete. This happens in from five to twenty-five per cent. of the hairs removed, and depends partly upon the skill of the operator and partly upon the direction of the hairs. In some cases the hair-shaft in the skin is so twisted that it is almost impossible to strike the papilla. Such hairs often require repeated removal before they are finally destroyed. The greatest success will usually be obtained on the upper lip and chin, while the hairs under the jaw will frequently return again and again to the great disappointment of both patient and physician. Partial failure should not discourage the operator. Persistence will surely be rewarded by success.

I may add that the older the growth of hair the more satisfactory will be the result. In young persons new hairs continually appear which sometimes lead the patient to think that the operation is unsuccessful and that all the hairs are returning. The fact of the continued growth of the hair should be explained to the patient before beginning the operation. In older persons, where the growth is complete, the new crop consists simply of those hairs which had not been destroyed and which grow out again. A second removal is followed by still fewer returns, and finally complete success is obtained. In younger individuals this period is longer deferred on account of the above-mentioned outgrowth of new hairs.

### 2. DESTRUCTION OF SMALL HYPERTROPHIES AND NEW FORMATIONS.

Warts, venereal condylomata, fibroids of the skin and pigmentary or vascular nævi can be readily removed by means of electrolysis without leaving disfiguring scars. The process is not painful. The same needle-holder above shown may be employed, but a stiff, steel needle should be used as the electrode. The base of the growth is transfixed in various directions and the current passed for a few minutes each time. The punctured tissues turn pale and slight frothing occurs around the needle. In most cases the growth, if a wart, mole or papillary growth, dries into a brownish crust and drops off in the course of a week or ten days, leaving a slightly pigmented spot which soon acquires the natural color of the surrounding skin.

Vascular nævi demand a slightly different procedure. When the growth consists of an ampulla-form dilatation of a vessel with radiating branches—the so-called "spider nævus"—the needle is plunged into the center of the dilated vessel and the current allowed to pass until the red or brown color has given place to a grayish discoloration. The current is then reversed for a few minutes, making the needle electrode positive. This insures a firmer clot in the vessel and prevents subsequent hemorrhage. In the course of a week the vessel is usually obliterated and the blemish has disappeared.

Flat vascular nævi (port wine mark) can be obliterated in a similar manner. It requires much patience on the part of physician and patient, however, if complete success is to be attained.

Hypertrophic scars and keloid growths can likewise be removed by electrolysis. Hardaway, of St. Louis, and Brocq, of Paris, have reported successful cases of keloid so treated. The disfiguring scars sometimes remaining after strumous and syphilitic ulcerations, or after vaccinations, can be much improved by the intelligent use of this method.

When large growths are operated upon it is frequently advisable to use a needle electrode connected with each pole of the battery and to plunge both needles into the tumor. In this way the resistance to the current is very much diminished and the result is more rapidly obtained. Care must be taken, however, to limit the electrolytic action to the pathological tissue, unless the growth is malignant, when a portion of the normal tissue should also be destroyed to prevent recurrence.

### 3. ABSORPTION OF LARGER NEW FORMATIONS BY ELECTROLYSIS.

The want of success with the usual methods of treatment of goitre should make any addition to our therapeutical resources against this disease desirable. The number of cases on record, which are reported to have been cured by electrolysis, is about sixty. Chvostek, Althaus, Gherini and Amory have reported successful cases. In two cases in which I employed the method great improvement was obtained. One of these was a girl of fourteen in whom the tumor produced interference with respiration on account of its size. The application of a current through the tumor without puncture resulted in diminishing the circumference of the neck one inch after twelve sittings. The relief to the breathing was so decided by this time that the patient discontinued her visits and the treatment was suspended. After an interval of ten months there has been no recurrence of the troublesome symptoms, although the thyroid is still much enlarged. Amory treats goitre by puncturing the gland with the needle used as the negative electrode, while the positive is placed on an indifferent point. Chvostek's method was that used by me in the case above referred to. The ordinary sponge electrodes are placed upon the skin on each side of the tumor and a current passed, varying the position of the electrodes and the direction of the current frequently. A sitting should last from ten to fifteen minutes.

Fibroid tumors of the uterus have recently been successfully treated by electrolysis. A French gynecologist, Apostoli, has been especially active in developing the method and bringing its merits before the profession. Within the last year a large number of communications have been made by practitioners of eminence, among whom Keith, Mundé and Engelmann may be especially mentioned. I have personal knowledge of a number of cases in which the method has been successfully used. The instruments needed are an insulated needle electrode connected with the negative pole (Fig. 2), and a large flat mass of moistened clay

FIG. 2.—APOSTOLI'S ELECTRODE.

FIG. 3.—APOSTOLI'S ABDOMINAL ELECTRODES.

(Fig. 3), to be used as the positive electrode. This is thoroughly softened and then placed upon the abdomen, pressing it well down upon the skin so as to insure perfect contact and reduce the resistance to the current as much as possible. Where the current is diffused over a large surface of skin, as by the use of this electrode very high currents can be employed. From 50 to 200 milliampères are frequently used in the treatment of uterine tumors by this method. The needle, connected as negative electrode, is plunged through the abdominal wall into the tumor and the circuit is then completed. The pain from the puncture can be prevented by a preceding hypodermic injection of cocaine. If the puncture has been made under antiseptic precautions, there is little fear of peritonitis. The uninsulated portion of the needle should be completely buried in the tumor. The current may be allowed to pass for ten minutes when the circuit is broken, the needle withdrawn and the clay electrode removed. Should pain or fever follow, an ice bag to the abdomen will soon relieve these symptoms. Usually some diminution in the growth is manifest in a few days. The operation may be repeated at weekly intervals until absorption has taken place.

In cases where hemorrhage is the most pronounced symptom of the growth, or in non-puerperal hemorrhage from any cause, a different method is pursued. Here an insulated sound (Fig. 4)

FIG. 4.—INTRA-UTERINE ELECTRODE.

is used as the positive electrode and introduced into the uterine cavity, while the clay electrode above described is used on the abdomen as a negative electrode.

Pelvic exudations can also be made to disappear under the influence of the electrolytic current. The exudation may either be punctured through the vagina by a needle used as negative electrode, or a vaginal electrode (Fig. 5) may be placed against the tumor and a large electrode of clay or of wire gauze, covered with absorbent cotton, over the abdomen. In the latter case the direction of the current may be varied during the sitting so as to send it in both directions.

FIG. 5.—INSULATED ELECTRODE FOR VAGINA.

### 4. STRICTURE.

The demonstration of the disruptive effect of the electrolytic current upon organic tissues led several surgeons to employ it for the removal of strictures, due to inflammatory new formations. Mallez and Tripier first reported successful cases, and within the past four or five years many observations have been placed upon record which leave no room for doubt that the practice gives successful results. Dr. Robert Newman, of New York, has been the most prominent advocate of the method. My own experience has convinced me that in electrolysis properly performed we have a method by which urethral stricture can be easily, painlessly and permanently cured. That all strictures of the urethra will be cured by this treatment is not claimed. It would be as irrational to subject all strictures to the electrolytic treatment as it would be to claim external urethrotomy as the exclusive remedy. Many strictures will be dilated, cut from within or without, in the future as in the past, but the treatment by electrolysis has won for itself a place in genito-urinary surgery which it will maintain. The evidence in its favor is too strong to be ignored. Ridicule, indifference, or denunciation from those who have failed to give the method fair trial, will be alike ineffective in causing its abandonment.

FIG. 6.—SOUNDS FOR ELECTROLYSIS OF STRICTURE.

In stricture of the urethra insulated sounds made to correspond in size to one of the three metrical systems used by genito-urinary surgeons are used as the negative electrode. The illustration (Fig. 6) shows several examples of these sounds. The ordinary sponge-covered rheophore is usually used as the positive electrode.

The method of performing the operation is first to measure the stricture with the urethra-meter of Gross, or Otis, or by the olive-pointed sounds. An insulated sound two or three sizes larger than the stricture will admit is then attached to the negative conducting cord of the battery and passed into the urethra until the stricture is reached. With the sponge electrode on the thigh or abdomen a current of from four to eight milliampères is passed through the circuit. It will be found that in a few minutes the sound will slip through the stricture without any effort on the part of the operator. In fact, the gentlest pressure only should be used in passing the stricture. I have seen the mere weight of the sound sufficient to overcome the obstruction after the passage of the current had continued for a short time. After the stricture is overcome (the size of the sound having been carefully noted) the patient is allowed an interval of several days, from five to eight being sufficient, before the second sitting. Then a sound two or three sizes larger than that first used should be passed through the obstruction in a similar manner. During the interval the urethra should not be irritated by the constant passage of sounds or measuring apparatus "to see whether the operation has accomplished anything." Common sense and good judgment are as necessary in the employment of electrolysis as in the handling of any other therapeutic resource. So far as my personal observation extends, strictures in the anterior portion of the urethra, where the obstruction can be exactly localized, and where the element of spasm can be largely excluded, offer the best cases for this method of treatment and the most satisfactory results. I have treated, however, a number of strictures in the post-penile portion of the urethra where the results were all that could be expected or desired.

The treatment of stricture of the rectum by gradual dilatation or linear proctotomy is notoriously unsatisfactory. All surgeons admit the inefficiency of the first method and the danger of the second. In electrolysis we have here a safe and apparently efficient method of treatment. I cannot refrain from citing here a case which came under my notice some time ago. The patient was a married woman, twenty-six years of age, who had contracted syphilis in her sixteenth year. This infection left a reminder in a stricture of the rectum, which, after ineffectual attempts at dilatation by means of bougies, was cut posteriorly by a surgeon of skill and experience. Subsequent dilatation with bougies kept the channel open for a time, but the pain and inconvenence of this treatment caused its neglect and final abandonment. After the death of the surgeon who had performed the proctotomy, the patient came into my hands. Upon consultation with my friend, Dr. S. T. Earle, electrolysis was tried at my suggestion, the treatment being thoroughly carried out by Dr. Earle. At the first examination a dense stricture was found, through which nothing larger than a fine silver probe could be carried. Only liquid stools could be passed with great pain and straining. A rectal injection was out of the question, as the smallest nozzle of the syringe would not go through the strictured portion of the bowel. The patient had almost given up all hope of recovery, and resolutely rejected a repetition of the operation of proctotomy, which was suggested to her by both Dr. Earle and myself before the idea of trying electrolysis had occurred to me. After three months treatment by electrolysis an electrode two and a half inches in circumference could be introduced beyond the stricture without giving pain; the patient had normal stools and was very much improved in general condition. *No constitutional or local anti-syphilitic treatment* was given during this time in order to avoid any fallacious conclusions as to the value of the electrolysis. But it is well known how utterly useless specific treatment is in old syphilitic stricture of the rectum. Even, therefore, if anti-syphilitic treatment had been given in this case, the conclusion that the electrolysis is a success would not be invalidated.

The method of operating in this malady is somewhat similar to that in urethral stricture. An insulated rectal electrode (Fig. 7)

FIG. 7.—RECTAL ELECTRODE.

is made to engage in the stricture, and the circuit closed by placing the positive electrode over the sacrum, abdomen or thigh, or, in the case of a female, in the vagina. In the case above cited a large mass of inflammatory (?) infiltration, situated in the recto-vaginal septum above the stricture also disappeared under the influence of the electric current.

Stenosis of the cervix uteri, which has given rise to so much discussion between the advocates of metrotomy and dilatation, can be more easily and probably more effectively treated by electrolysis. I have no personal experience with the method in this common affection, but several professional friends, devoted especially to gynecological practice, speak highly of its efficiency. Engelmann has also placed on record his successful experience with the method.

It seems to me that electrolysis has a future of great usefulness in the treatment of strictures of the nasal duct and stenosis of the eustachian tube. An early resort to electrolysis in the latter affection would, in my opinion, very much reduce the large number of cases of incurable deafness. Of course, the cases should be carefully selected in order not to bring the method into disrepute. When hypertrophy, or sclerosis of the lining membrane of the tympanic cavity, and the consequent interference with the normal mobility of the ossicles has taken place, it is manifestly out of the question to hope for much improvement from merely rendering the tube pervious. Before these secondary pathological conditions have been established, however, the employment of electrolysis is not only rational, but full of promise.

*611 N. Calvert St., Baltimore, Md.*

## Figures and Tables

**Fig. 1. f1:**
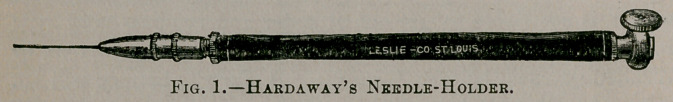


**Fig. 2. f2:**
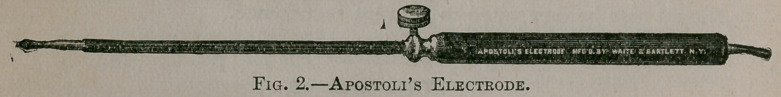


**Fig. 3. f3:**
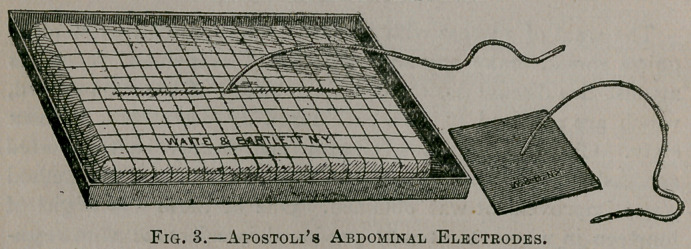


**Fig. 4. f4:**
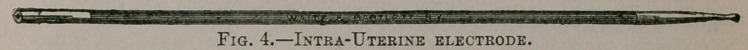


**Fig. 5. f5:**
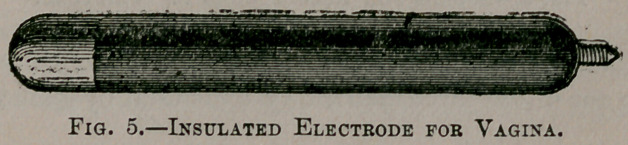


**Fig. 6. f6:**
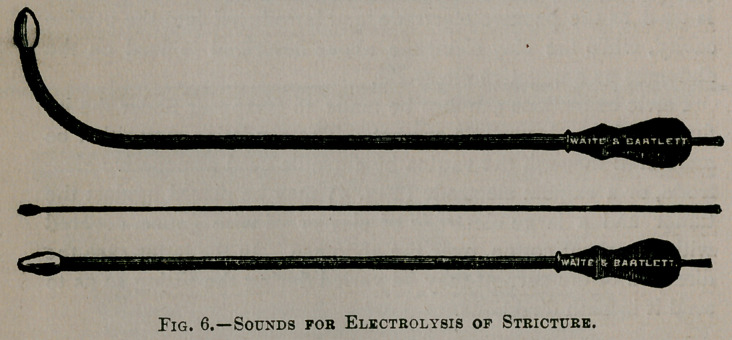


**Fig. 7. f7:**